# Malaria in pregnancy: a passive surveillance study of pregnant women in low transmission areas of Colombia, Latin America

**DOI:** 10.1186/s12936-016-1125-9

**Published:** 2016-02-05

**Authors:** Mary Lopez-Perez, M. Andreína Pacheco, Lucía Buriticá, Ananias A. Escalante, Sócrates Herrera, Myriam Arévalo-Herrera

**Affiliations:** Caucaseco Scientific Research Center, Cali, Colombia; Institute for Genomics and Evolutionary Medicine (igem), Temple University, Philadelphia, PA USA; Department of Biology, Temple University, Philadelphia, PA USA; Faculty of Health, Universidad del Valle, Cali, Colombia

**Keywords:** Malaria, Pregnancy, Colombia, *Plasmodium*, Antibodies, Microsatellite repeats

## Abstract

**Background:**

Malaria causes a significant burden in highly endemic areas where children and pregnant women are more susceptible to severe disease and death, however, in low transmission settings malaria in pregnant women is less frequent. The aim of this study was to provide information of clinical profile, anti-parasite host immune responses and parasite genotyping of pregnant women with malaria in low endemic areas of Colombia.

**Methods:**

This was a descriptive study conducted through passive surveillance in 1328 individuals from three endemic areas of Córdoba, Nariño and Chocó departments between 2011 and 2013. Trained physicians confirmed the pregnancy status and recorded clinical and epidemiological information. Haematological parameters, as well as hepatic and renal function, anti-malarial antibodies and parasite genotypes were evaluated.

**Results:**

A total of 582 women presented with malaria infection, 34 of whom were pregnant (5.8 %), and most were infected by *Plasmodium falciparum* (n = 24). In 44 % (n = 15) of the women, the infection occurred during the first half of pregnancy. Although uncomplicated disease and parasitaemia ≤20,000 parasites/µL were common (n = 31), three women (8.8 %) infected by *P. falciparum* were classified as severe cases. Mild to moderate anaemia (68 %) and mild thrombocytopaenia (41 %) were the most frequent blood alterations and in four women acute renal failure was observed. Six women presented a second malaria episode during pregnancy mainly caused by *P. vivax* (n = 5), although no direct evidence of relapse was found by genotyping. Two out of the six women presenting a second malaria episode had severe malaria. A low prevalence of specific anti-parasite antibodies was found. Microsatellites indicated that all *P. vivax* infections involved multiple lineages whereas all but one *P. falciparum* infections harboured single genotypes.

**Conclusions:**

Most malaria infected pregnant women displayed uncomplicated malaria, although a few of them with a second malaria episode presented an increased risk of severe malaria which appeared to be associated with malaria transmission intensity and not with levels of anti-parasite antibodies. The effects of severe malaria in both mother and fetus warrant future studies in low transmission settings.

**Electronic supplementary material:**

The online version of this article (doi:10.1186/s12936-016-1125-9) contains supplementary material, which is available to authorized users.

## Background

Malaria in pregnancy (MiP) is one of the leading causes of maternal and child morbidity and mortality worldwide, mainly in high endemic areas [1–3]. About 125 million women are at risk of acquiring malaria infections during pregnancy [2], mainly in Africa where *Plasmodium falciparum* is the predominant malaria parasite. Outside Africa, *Plasmodium vivax* infections are predominant and every year about 90 million pregnant women are exposed to the risk of infection mostly in the Asia–Pacific region only [2, 3] and about three million in Latin America [2].

Malaria infection during pregnancy is associated with a broad spectrum of clinical manifestations ranging from acute uncomplicated to severe cases and death of both mothers and neonates [1, 4–6]. A higher risk of severe anaemia has been reported in *P. vivax* infected multigravidae [7–9]. Furthermore, *P. vivax* in Latin America has been associated with severe malaria [10–12], moderate-to-severe anaemia and low birth weight [8]. From ~390,000 malaria clinical cases reported in Latin America [13], low frequency of MiP cases (10 %) [8, 10–12, 14] with moderate-to-high severe cases (4–9 %) and low mortality (0–0.2 %) [8, 11, 12] have been observed. A comparable prevalence of malaria-associated deaths has been reported in the Thai-Myanmar border where the frequencies of annual fatal cases in pregnant women for *P. falciparum* and *P. vivax* were estimated as 0.28 % (12/4158) and 0.023 % (1/4298), respectively [6].

Given its public health importance, understanding the clinical manifestations of malaria during pregnancy has been the focus of several investigations. In particular, there is evidence indicating that the clinical presentation of the disease in pregnant women is modified by the immune status of the patient [5] as well as by the presence of multiple parasite genotypes [15, 16]. However, such associations may vary across the disease’s broad geographic distribution. Although studies in African pregnant women infected with *P. falciparum* found that levels of specific antibodies rapidly decrease during pregnancy rendering women more susceptible to MiP complications [17]; studies in Thailand, where both *P. falciparum* and *P. vivax* co-exist, have not shown such a clear pattern. One such study found a high variability of antibodies over time and its maintenance was associated with the number of malaria infections [18]. Regarding the proposed association between presence of multiple parasite genotypes for *P. vivax* and *P. falciparum* infections and pregnant women, the issue remains unsolved due to the paucity of studies that include parasite genetics [15, 16, 19, 20].

This study was performed in Colombia, a South American country with a low average malaria transmission. Colombia is the third contributor of malaria cases in Latin America, after Brazil and Venezuela [13] with approximately 10 million people currently living in areas with risk of malaria transmission. It is estimated that at least one million women of reproductive age (15–49 years) live in three of the most malaria endemic areas (Córdoba, Nariño and Chocó) of the country [21]. The aim of this study was to provide information from endemic areas in Latin America where, unlike other regions with low malaria transmission intensity [3, 6, 7, 9], there are few studies on MiP [10–12, 14]. Furthermore, Latin America MiP studies have not reported levels of humoral immune responses and only one study included parasite genotyping [19], thus the frequency of relapses in pregnant women or the role played by the presence of multiple parasite genotypes, if any, are not usually addressed. In this study, the clinical profile of a group of pregnant women acutely infected with *P. falciparum* and *P. vivax* is reported.

## Methods

### Study design, sites and ethical issues

This is a descriptive study in Colombian pregnant women carried out using data collected between 2011 and 2013 as part of a passive surveillance study conducted in Tierralta (Córdoba department), Quibdó (Chocó department) and Tumaco (Nariño department) Colombia, to characterize the malaria clinical profile [22]. These endemic regions have distinct malaria transmission intensities and parasite distribution where *P. vivax* was more frequent in Tierralta (~85 %), whereas *P. falciparum* was more frequent in Tumaco (~79 %) and Quibdó (~70 %) [23]. The average annual parasite incidence (API) between 2011 and 2013 in those sites was 6.7, 10.3 and 25, respectively.

Of a total of 1328 patients with symptomatic malaria enrolled in these three sites, 34 were pregnant women. The study protocol was previously approved by the Institutional Review Board (IRB) affiliated to the Malaria Vaccine and Drug Development Center (MVDC, Cali). Declaration of free willingness to participate in the study and written informed consent (IC) or an informed assent (IA) in the case of women <18 years of age were obtained from each participant. After blood samples were drawn, the local health provider treated all malaria infected women using the Colombian protocol [24]. Women infected with *P. falciparum* received artemether plus lumefantrine (orally, twice a day over 3 days), whereas those infected with *P. vivax* were treated only with chloroquine (orally, 25 mg/kg provided in three doses). Severe malaria cases were treated at the hospital with intravenous artesunate (2.4 mg/kg time 0, with repeat doses at 12 and 24 h), followed by oral artesunate (once a day to complete 7 days). All women were asked to return 7 days after treatment for thick blood smear (TBS) control and at any moment if malaria symptoms were presented. Intermittent preventive treatment (IPTp) was not provided, because it is not included in the Colombian national policy.

### Case definition

Patients were classified as suffering from uncomplicated or severe MiP according to the clinical and laboratory criteria defined by the WHO [25] and the Colombian MoH guidelines [24]. The latter are more conservative in some definitions based on previous evidence i.e. severe anaemia (Hb <7 g/dL), renal dysfunction (serum creatinine >1.5 mg/dL), severe thrombocytopaenia (≤20,000 platelets/µL) and hyperparasitaemia (>50,000 parasites/μL). Uncomplicated MiP was defined as a clinical malaria case with the presence of *Plasmodium* spp. in the peripheral blood without severity criteria. Severe MiP was defined as one or more of the clinical or laboratory parameters previously described by either the Colombian or WHO guidelines [24, 25], regardless of the malaria parasite species. Trained physicians of the study staff completed a standard clinical evaluation and physical examination in all women and then blood samples were obtained. In all women, the pregnancy was confirmed using pregnancy dipstick test (Abon Biopharm Company, China). The gestational age was measured calculating the days since the beginning of the last menstrual period.

### Laboratory tests

Whole blood (15 mL) was collected by venipuncture at the time of enrolment before the anti-malarial treatment was provided. Malaria diagnosis was performed by microscopic examination of Giemsa-stained TBS, which were independently examined by two experienced malaria microscopists. Parasite density (parasites/µL) was estimated by counting the number of parasites per 200 leukocytes and normalized using the actual leukocyte counts of each woman [(number of parasites × leukocyte counts)/200 leukocytes]. Malaria parasite species was retrospectively confirmed in all samples by real time PCR, as described elsewhere [22]. Automated complete blood cell counts (KX-21 N, Sysmex, Japan) and urine analysis (dipstick and microscopic) were performed at each point-of-care (POC). Blood chemistry profiles in frozen sera were analysed using commercial kits in a reference laboratory in Cali (Asoclinic Ltda) following manufacturer’s instructions. Renal (creatinine and blood urea nitrogen, BUN) and hepatic function (total bilirubin and aminotransferases, ALT and AST) parameters were analysed spectrophotometrically (BTS-350 Chemistry Analyser, Biosystems SA, Spain) using commercially available kits (Biosystems SA, Spain).

### ELISA and IFA tests for specific malaria antibodies

Specific IgG anti-malarial antibodies in serum were determined both by immunofluorescent antibody (IFA) and enzyme-linked immunosorbent assay (ELISA) tests. Because both *P. vivax* and *P. falciparum* are present in the study areas, sera from all patients were analysed for antibodies to both parasite species. IFA tests were performed using *P. vivax* and *P. falciparum* blood stages antigen preparations derived from infected patients or from in vitro cultures respectively as previously described [26]. Antibody titers were estimated using twofold serial dilutions of the test sera starting at 1:20. ELISA test was used to determine the presence of total IgG specific to the *P. vivax* circumsporozoite protein (*Pv*CS, N-terminal fragment), the merozoite surface protein-1 (*Pv*MSP-1, r200L recombinant protein) as well as the response against *P. falciparum* CS (*Pf*CS, N-terminal) and MSP-1 (*Pf*MSP-1, 19 kDa recombinant protein) as described elsewhere [27]. Optical Density (OD) at 405 nm was measured using a BioTek ELISA Reader (BioTek, Winooski, VT). Cut-off values were calculated as three SD above the mean OD value of negative control sera. A sample was considered positive when the OD at 1:200 dilution was higher than the cut-off value. Results were expressed as a reactivity index (RI), defined as OD values of test sample divided by the cut-off value.

### DNA extraction and microsatellite (STRs) genotyping

Whole blood samples collected with EDTA anticoagulant were used for extraction and purification of genomic DNA using the QIAamp DNA Blood kit according to manufacturer’s instructions (Qiagen Inc, CA, USA). The DNA was eluted and stored at −20 °C until use. Genotyping was performed using fluorescently labeled PCR primers. Those with low parasitaemia were amplified by whole genome amplification using the REPLI-g Mini Kit (Qiagen Inc, CA, USA). A set of 10 standardized STRs loci for *P. vivax* and nine for *P. falciparum* selected from an extensive pool of choices that have been explored [28] were used. In the case of *P. vivax,* the following loci were included in the analyses: MS2, MS5, MS6, MS15 [29] and 14.185, 7.67, 8.332, 2.21, 10.29, 8.332 [30]. Loci POLYa, TAA60, ARA2, Pfg377, TAA109, TAA81, TAA42-3, TA40 and PfPK2 were amplified for *P. falciparum* [31]. Fluorescently labelled PCR products were separated on an Applied Biosystems 3730 capillary sequencer and scored using Gene Marker v1.95 (SoftGenetics LLC). The presence of one or more additional alleles at a particular locus was interpreted as a co-infection with two or more genetically distinct clones in the same isolate [28].

### Statistical analysis

Study data were collected and managed using REDCap (Nashville, Tennessee, USA) electronic data capture tool [32]. Data were analysed with the statistical software MATLAB^®^ 2013a (The MathWorks, Inc., Natick, Massachusetts, USA). The nominal variables were analysed using descriptive statistics. The Mann–Whitney U test was used to compare two groups and Wilcoxon signed rank test for paired data. Spearman’s rank correlation (r_s_) was used to assess the correlation between numeric variables. Fisher’s exact test was used to compare proportion differences. A p value <0.05 was considered statistically significant.

## Results

### Epidemiological characteristics of the pregnant women

From 1328 subjects infected with malaria, 582 were women and 34 of them were pregnant (5.8 %). Most patients were ≤25 years of age (76 %; range 14–41 years) and a high proportion of them (n = 15; 44 %) became infected in the first half of pregnancy, seven of them during the first trimester. Afro-descendants (n = 19; 56 %) and mestizos (n = 13; 38 %) were the most frequent ethnic groups and the remainder corresponded to indigenous population. In total, 62 % (n = 21) of women had resided in the malaria-endemic region (>2 years, median 4.5 years) and 88 % (n = 30) were working at home. Half of the women self-reported previous lifetime malaria episodes and in eight women this happened in the previous year (Table 1).

**Table 1:** Characteristics of pregnant women and malaria history

	*Plasmodium falciparum* (n = 24)	*Plasmodium vivax* (n = 10)	P value^b^
	Median (IQR)^a^	Median (IQR)	
Age (years)	20 (17–28)	19 (17–23)	0.344
Weeks of gestation	24 (20–30)	*16 (8*–*23)*	*0.033*
Time of residence (years)	*6 (1.5*–*15.5)*	0.3 (0.1–7.5)	*0.026*
Number of previous malaria episodes	2 (1–4)	3 (1–4)	0.681
Days of illness	5 (3–8)	4 (3–7)	0.623

Significant data are highlighted in italics

^a^ *IQR* interquartile range

^b^p value using the Mann–Whitney U test between *P. falciparum* and *P. vivax*

### Malaria diagnosis and clinical manifestations

All malaria infections were confirmed by real time PCR as *P. falciparum* or *P. vivax* monoinfection, with *P. falciparum* as the most prevalent (n = 24; 71 %, Table 1). Most cases (91.2 %) presented with low to moderate parasitaemia, ≤20,000 parasites/µL. The median *P. vivax* parasitaemia (7547 parasites/µL; IQR 4, 499–10,721; min–max 1740–15,100) appeared higher than in *P. falciparum* infections (2509 parasites/µL; IQR 869–10,083; min–max 355–36,919). Such difference was not statistically significant.

**Fig. 1:**
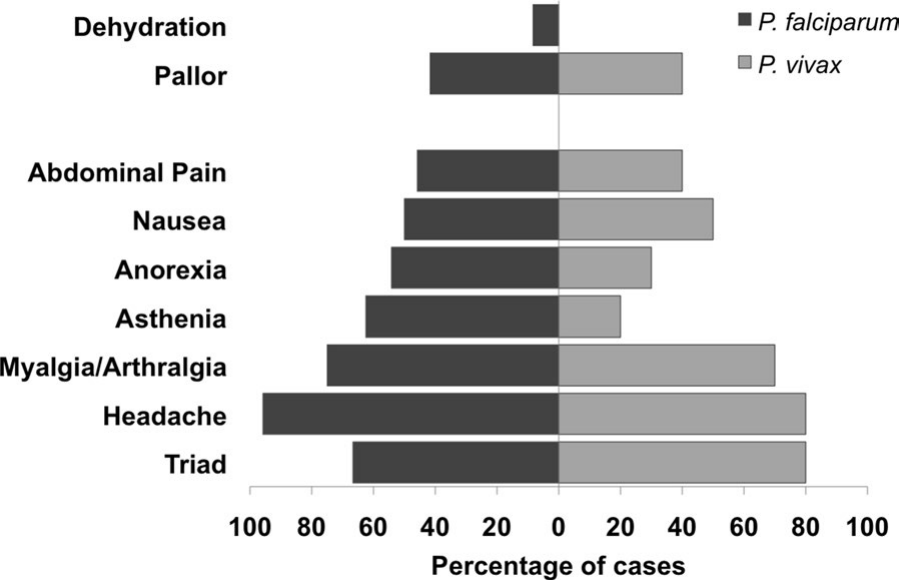
Frequency of clinical manifestations in *P. falciparum* and *P. vivax* infections. Percentages of women presenting every clinical manifestation are shown. All women reported more than one. Triad corresponds to women presenting fever, chills and sweating. No significant differences were observed

Seven women (21 %) attended at the POC for diagnosis within 48 h after symptoms onset (range 0–22 days) and only four women reported >15 days of illness (Table 1). At day 7 all women had negative TBS results. According to the clinical and laboratory parameters, uncomplicated malaria was the most frequent clinical syndrome (91.2 %). However, after evaluation at the POC 19 of the 34 patients were referred to a hospital for observation and treatment. The classical triad of fever, chills and sweating together with headache was reported by 24/34 women (71 %). Myalgia/arthralgia (74 %), asthenia (50 %), nausea (50 %), anorexia (47 %) and abdominal pain (44 %) were frequently reported independently of the malaria parasite species (Fig. 1). On physical examination, pallor (41 %), fever (32 %) and abdominal pain on palpation (21 %) were observed. Notably, seven women (four with *P. falciparum* and three with *P. vivax*) reported one or more of the following symptoms associated with pregnancy: reduction of foetal movement, preeclampsia signs or abdominal and pelvic pain (sudden and intense) without signs of preterm delivery.

### Haematological alterations

There were no significant differences for haematological parameters induced by the two parasite species (Table 2). Mild-to-moderate malaria-related anaemia was observed in 68 % of the studied women, with only one who suffered a second malaria episode during the current pregnancy, presenting severe anaemia (Hb <7 g/dL). Six women (18 %) presented microcytic anaemia. Haemoglobin levels remained significantly associated with age regardless of the parasite species (r_s_ = 0.358; p = 0.038). Thrombocytopaenia (<150,000 platelets/µL) was found in 41 % (n = 14) of the women, but only three of them had <100,000 platelets/µL. A negative correlation was found between platelets counts and parasitaemia (r_s_ = −0.557; p < 0.001), but it was not associated with parasite species, age or weeks of gestation (data not shown). Mild leukopaenia (18 %), lymphopaenia (15 %) and neutropaenia (5 %) were observed mainly in *P. falciparum* infected women.

**Table 2:** Laboratory parameters in pregnant women with malaria

Laboratory parameters	*P. falciparum* (n = 24)	*P. vivax* (n = 10)	p value^b^
	Median (IQR)^a^	Median (IQR)	
Haemoglobin (g/dL)	10.1 (9.1–11.0)	10.6 (9.2–11.5)	0.461
Platelets count (×10^3^/µL)	163 (132–242)	143 (124–197)	0.281
Leukocytes (×10^3^/µL)	5.6 (4.6–6.8)	6.2 (5.9–7.6)	0.073
Neutrophils (×10^3^/µL)	3.8 (2.9–4.7)	3.8 (3.6–4.6)	0.545
Lymphocytes (×10^3^/µL)	1.5 (1.1–2.0)	2.4 (1.4–3.1)	0.054
Total bilirubin (mg/dL)	0.6 (0.2–0.8)	0.6 (0.3–1.3)	0.493
ALT (U/L)	13.0 (10.0–20.0)	13.5 (10.5–17.0)	0.784
AST (U/L)	25.0 (16.0–33.0)	22.5 (17.5–27.5)	0.318
Creatinine (mg/dL)	0.7 (0.7–0.9)	0.7 (0.6–0.8)	0.290
BUN (mg/dL)	8.0 (6.0–11.0)	8.5 (7.0–12.0)	0.317

*BUN* blood urine nitrogen, *ALT* alanine aminotransferase, *AST* aspartate aminotransferase

^a^ *IQR* interquartile range

^b^p value using the Mann–Whitney U test between *P. falciparum* and *P. vivax*

### Hepatic and renal alterations

Median values of renal and hepatic function laboratory parameters were similar in both parasites species (Table 2), although mild (10 %) to moderate (16 %) hyperbilirubinaemia (total bilirubin range 1.0–2.1 mg/dL) was observed particularly in *P. vivax* infections. Hepatic enzymes (ALT and AST) were altered in 21 % (n = 7) of women, all of them infected with *P. falciparum*. Proteinuria (61.8 %), ketonuria (29.4 %) and leukocyturia (23.5 %) were observed in women infected with either parasite species. Acute renal failure of pre-renal origin (BUN: creatinine >20) was found in four women, of whom two had also proteinuria and choluria. Using standard criteria in pregnancy [33], eight women (24 %) had alterations in the serum creatinine levels (>0.8 mg/dL) and six (18 %) in the BUN levels (>12 mg/dL), mostly infected by *P. falciparum,* 6/8 and 4/6, respectively.

### Second malaria episode during gestation

As described above, patients were asked to return to the POC if malaria symptoms recurred. A total of six women (one with *P. falciparum* and five with *P. vivax*) presented with a second malaria episode by the same parasite species, even after receiving treatment for asexual parasite stages in the first episode; however primaquine was not administered for *P. vivax* due to its contraindication during pregnancy. Since the follow-up was passive, new episodes, complications or self-treatment in some of the women cannot be ruled out.

**Fig. 2:**
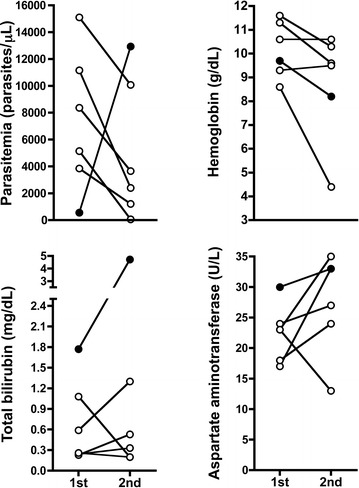
Laboratory parameters for the first and second malaria episodes. Data of women infected with *P. vivax* (n = 5; *open circles*) and *P. falciparum* (n = 1; *closed circle*) are shown. Statistical differences between both episodes were calculated using the Wilcoxon signed rank test; all of them were not significant (p > 0.05)

Median time between episodes was 59 days (IQR: 37–158; range 27–200 days) and patients reported similar signs, symptoms, and days of illness before consultation (median 4 days) but lower parasitaemia than in the first episode (median 6759 vs 3030 parasites/µL, respectively; Fig. 2), except for the woman infected by *P. falciparum* presenting higher parasitaemia in the second episode. All six women presenting with a second episode were anaemic with a lower Hb than in the first episode (Fig. 2). Most patients presented normal values for other laboratory parameters (Table 3).

**Table 3:** Parameters in the six pregnant women presenting a second malaria episode

Parameters	First episode (n = 6)	Second episode (n = 6)	p value^b^
	Median (IQR)^a^	Median (IQR)	
Weeks of gestation	18 (10–29)	*33 (23*–*38)*	*0.031*
Days of illness	4 (2–13)	4 (2–6)	0.625
Parasitaemia (parasites/µL)	6759 (2207–13,130)	3030 (641–11,506)	0.438
Haemoglobin (g/dL)	10.2 (8.9–11.5)	9.6 (6.3–10.5)	0.079
Platelets count (×10^3^/µL)	155 (133–306)	167 (131–264)	0.844
Leukocytes (×10^3^/µL)	6.2 (5.1–8.6)	7.5 (5.4–9.3)	0.688
Neutrophils (×10^3^/µL)	3.8 (3.7–5.1)	4.7 (3.6–7.1)	0.313
Lymphocytes (×10^3^/µL)	2.4 (1.2–3.4)	2.0 (1.2–2.7)	0.563
Total bilirubin (mg/dL)	0.43 (0.24–1.43)	0.43 (0.20–3.01)	0.374
ALT (U/L)	12.5 (9.5–19.0)	11.5 (6.5–16.0)	0.346
AST (U/L)	23.0 (17.5–27.0)	30.0 (18.5–34.0)	0.231
Creatinine (mg/dL)	0.7 (0.6–0.7)	0.7 (0.5–0.8)	1.000
BUN (mg/dL)	8.5 (6.5–10.0)	8.5 (7.0–11.0)	0.563

*BUN* blood urine nitrogen, *ALT* alanine aminotransferase, *AST* aspartate aminotransferase

^a^ *IQR* interquartile range

^b^ p value using the Wilcoxon signed rank test

### Severe malaria

Three *P. falciparum* cases (3 of 34 women = 8.8 %) were classified as severe malaria during the first episode: two women developed prostration and required hospitalization, and one had severe thrombocytopaenia (20,000 platelets/µL) without spontaneous bleeding. The three women also presented moderate anaemia (mean Hb 8.7 ± 0.1 g/dL). All women received anti-malarial and supporting treatment according to severity of infection as recommended by Colombian guidelines [24].

In addition, two 15 year-old women presenting with a second malaria episode during pregnancy, were severe. One woman with*.P. falciparum* infection (12,932 parasites/µL) presented hepatic dysfunction with hyperbilirubinaemia (total bilirubin 4.7 mg/dL) and clinical jaundice, while another with *P. vivax* (3655 parasites/µL), presented severe anaemia (Hb 4.4 g/dL) with general pallor without co-incidental infections. No deaths were reported.

### Antibody responses

**Fig. 3:**
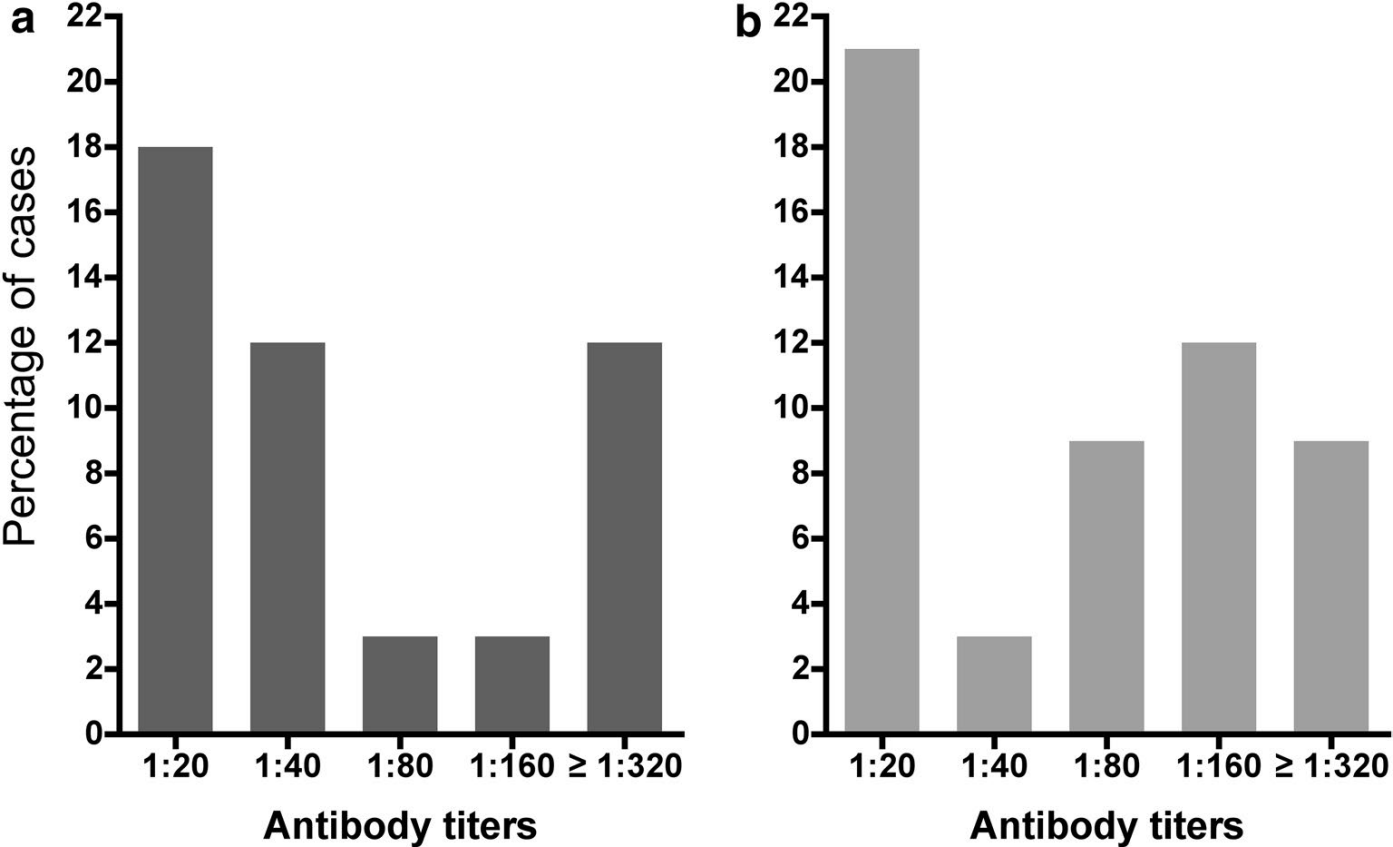
Seroprevalence of IgG antibodies against whole blood stages tested by IFA. Frequency of IgG titers against (**a**) *P. falciparum* and (**b**) *P. vivax*

**Fig. 4:**
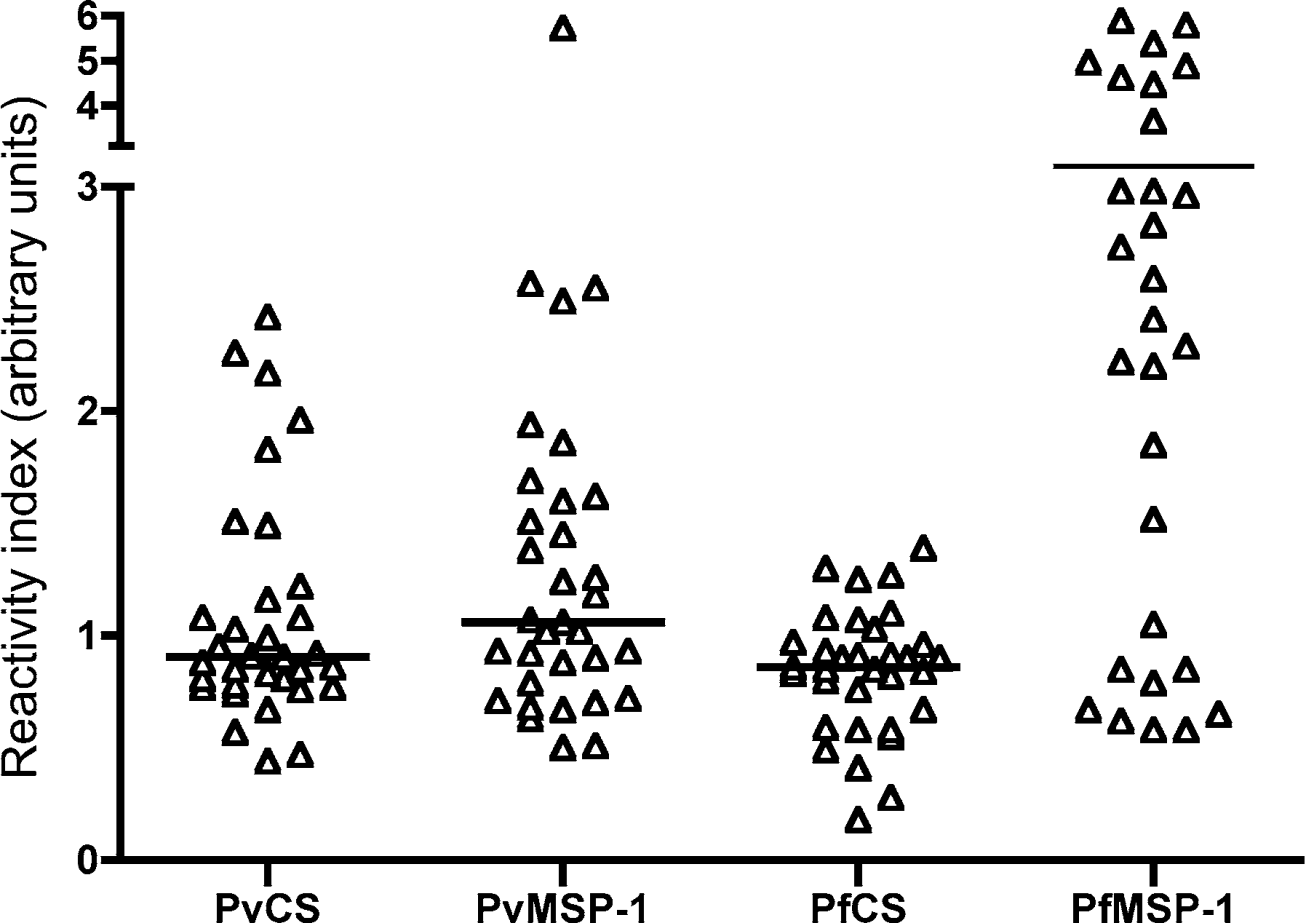
Antibody response against *Plasmodium* antigens during patent malaria infection. Response against *Pv*CS, *Pv*MSP-1, *Pf*CS and *Pf*MSP-1 was determined by ELISA. Values are expressed as reactivity index (RI) defined as sample OD at 1:200 serum dilutions divided by the cut-off value. *Solid lines* show the median RI in each group

**Fig. 5:**
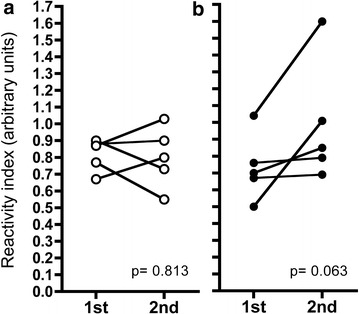
Antibody response against *P. vivax* antigens in women with two malaria episodes by *P. vivax*. Antibody response against (**a**) *Pv*CS and (**b**) *Pv*MSP-1 was determined by ELISA. Values are expressed as reactivity index (RI) defined as sample OD at 1:200 serum dilutions divided by the cut-off value. Statistical differences between both episodes were calculated using the Wilcoxon signed rank test

A total of 47.1 % (n = 16) and 52.9 % (n = 18) pregnant women had antibodies to whole *P. vivax* and *P. falciparum* blood stages by IFA test respectively and significant differences were observed between antibody specific parasite species (Table 4) with low antibody titers ranging between 1:20 and 1:640 (Fig. 3). When antibodies were determined by ELISA the frequency of responders for each antigen were low-to moderate: *Pv*CS (32.4 %), *Pv*MSP-1, (55.9 %), *Pf*CS (26.5 %) and *Pf*MSP-1 (73.5 %); 8.8 % (n = 3) and 47 % (n = 16) women were double positive for *P. vivax* and *P. falciparum* CS and MSP-1 antigens, respectively, however antibody levels displayed low titers (Fig. 4). Differences in reactivity (RI) between *P. falciparum* and *P. vivax* infected women were observed for *Pf*CS (median 0.90 vs 0.59; p = 0.023) and *Pf*MSP-1 (median 3.66 vs 1.29; p = 0.008), respectively. In contrast, the RI values for *Pv*CS and *Pv*MSP-1 were similar in *P. falciparum* and *P. vivax* infected women (median 0.91 vs 0.89 and 1.18 vs 0.80, respectively). In women who had two *P. vivax* episodes during pregnancy an increase in the RI for *Pv*MSP-1 during the second episode was observed (Fig. 5). Likewise in the woman infected with *P. falciparum* the RI for *Pf*MSP-1 increased (0.58 vs 6.81). No association was found between antibody titers and disease severity.

**Table 4:** Seroprevalence against *Plasmodium* whole blood-stages as assessed by IFA

Asexual stages of	*P. falciparum* (n = 24)	*P. vivax* (n = 10)	p value^a^
	n (%)	n (%)	
*P. falciparum*	*16 (66.7* *%)*	0 (NA)	*0.001*
*P. vivax*	9 (35.5 %)	*9 (90* *%)*	*0.008*

Most frequent and significant data are highlighted in italics

^a^ p value using the Fisher’s exact test between *P. falciparum* and *P. vivax*

### Microsatellite (STRs) genotyping

A total of 22/34 parasite samples including *P. falciparum* (14/24) and *P. vivax* (8/10) were analysed using a set of physically unlinked microsatellite loci (Additional file 1). Most of the *P. falciparum* infections were single clonal infections (13/14), with six genotypes in 14 samples and only one sample with two genotypes differentiated by one locus. In comparison, *P. vivax* was more diverse, with all eight samples with two or more genetically distinct lineages.

In the woman presenting two *P. falciparum* episodes, the same genotype was found in both episodes, whereas the two women who presented two *P. vivax* episodes had distinct genotypes with differences in at least three loci between episodes (Additional file 1). In the *P. falciparum* patient the second episode corresponded to a severe case.

## Discussion

Despite the study areas being among the most endemic regions of Colombia [23], the low MiP prevalence observed (34/582; 5.8 %) was expected as malaria prevalence has significantly decreased in the country during the last decade [34], probably due to significant efforts by the National Malaria Control Programme (NMCP) to decrease the malaria incidence in the frame of control programmes sponsored by the Global Fund for Aids, Tuberculosis and Malaria (GFATM). However, although reducing malaria transmission is important, it has recently been suggested that the concomitant reduction in naturally acquired immunity to malaria increases the risk of adverse consequences for infected pregnant women [17].

Previous studies carried out in Colombia (Córdoba and Antioquia) indicated a higher prevalence (9 %–14 %) of MiP [12, 35, 36] and similar results were reported from Brazil and Bolivia, where prevalence of MiP ranged between 4 and 12 %, mainly due to *P. vivax* [8, 11]. Indeed, as reported here severe malaria in pregnant women was not rare (3/34; 8.8 %), with figures similar to those reported in low transmission areas in Colombia (15/166; 9 %) [12] and Thailand (9/121; 7.4 %) [1]. Suggesting that malaria diagnosis and anti-malarial treatment are occurring late since only 21 % of studied women consulted within the 48 h after symptoms onset, with a median of 4 days. In a recent study conducted in Colombia, significant awareness of the importance of early diagnosis and prompt treatment was found, which are becoming more common. Late screening in MiP would also explain that several women developed alterations such as mild-to-moderate anaemia (23/34), thrombocytopaenia (14/34) and renal dysfunction of pre-renal origin (8/34), which could significantly contribute to the risk of complications and death of both mother and fetus by increasing the risk of preterm delivery, low birth weight and post-partum haemorrhages [3, 5]. Despite the absence of fatalities in this study, those findings highlight the importance of early diagnosis and prompt treatment to decrease the risk of complication in low transmission settings.

Importantly, younger maternal age (≤25 years) observed in 76 % of patients and early malaria infection (<20 weeks of gestation) in 44 %, appeared associated with clinical presentation and may to increase the risk of intrauterine growth restriction [37, 38]. A similar age in pregnant women with malaria has been previously reported in Colombia [12, 19, 36], which may be related to the malaria transmission intensity and social and civil conflicts in this region (Arévalo-Herrera et al, unpublished data), indicating an important target population for malaria control programmes. In this study, moderate anaemia was associated with all severe malaria cases (3/3) and the presence of a second malaria episode (6/6) as reported in *P. falciparum* infections [39]. Although *P. vivax* is more prevalent in Colombia, most MiP cases reported here were induced by *P. falciparum,* particularly in Tumaco and Quibdó, two areas where population is Afro-descendant with high prevalence of Duffy-negative individuals [40].

Previous exposure to malaria infection might have contributed to a higher percentage of uncomplicated cases. Between 47 and 53 % of women had antibodies against whole blood stages, which confirmed self-reported previous exposure to parasite. A previous study demonstrated the development of significant protection from clinical symptoms in young adults from the study regions exposed to experimental *P. vivax* infection [41], in addition, it has been suggested that multiplicity of infections may be involved in the gradual acquisition and maintenance of malaria immunity [15]. Most women infected with *P. falciparum* harboured single genotypes regardless of disease severity; those also reported longer residence in the endemic areas. In contrast, all infections with *P. vivax* involved multiple genotypes. Such differences between *P. falciparum* and *P. vivax* have been observed in many malaria endemic areas [42]. Although the small sample size precludes further analysis between multiple genotypes and severity association in MiP, recently it was found that having a multiclonal *P. vivax* infection is associated with severe malaria in Colombian patients [43]. In this study, women presenting with a second *P. vivax* episode displayed different genotypes suggesting a new infection. However, a relapse cannot be ruled out due to the restriction of using primaquine during pregnancy [44]. In contrast, the same *P. falciparum* genotype was found in the women presenting two episodes. The second episode occurred 27 days after the first one and was a severe case, but the parasite had wild-type allele of the *K13* gene associated with artemisinin-based combination therapy resistance [45], suggesting a late therapeutic failure to artemether plus lumefantrine in this woman. In Indonesia, dihydroartemisinin-piperaquine is the first line of treatment for both *P. falciparum* and *P*. *vivax* in pregnant women [46]; therefore, an alternative option could be evaluated and considered in Colombia in case of artemether plus lumefantrine failure. Finally, as a consequence of the high prevalence of severe MiP cases, this study suggests the need for a more careful medical follow up during pregnancy in malaria endemic settings.

## Conclusions

Prompt diagnosis, effective treatment, and partial acquisition of clinical immunity appear to be associated with the high prevalence of uncomplicated MiP. However, anaemia and acute renal failure observed warrant more detailed studies because of the potential deleterious effects on both mother and fetus. This study reinforces the need for a more careful medical follow up during pregnancy, including malaria diagnosis as part of antenatal care for all pregnant women residing in endemic areas, to decrease the risk of complications and to avoid multiple malaria episodes associated with greater malaria severity.

## Additional file

10.1186/s12936-016-1125-9 Parasite genotypes from pregnant women samples.

## Abbreviations

ALT: alanine aminotransferase
AST: aspartate aminotransferase
BUN: blood urea nitrogen
MiP: malaria in pregnancy
POC: point-of-care
RI: reactivity index
